# Diagnostic Value of Magnetocardiography to Detect Abnormal Myocardial Perfusion: A Pilot Study

**DOI:** 10.31083/j.rcm2510379

**Published:** 2024-10-23

**Authors:** Huan Zhang, Zhao Ma, Hongzhi Mi, Jian Jiao, Wei Dong, Shuwen Yang, Linqi Liu, Shu Zhou, Lanxin Feng, Xin Zhao, Xueyao Yang, Chenchen Tu, Xiantao Song, Hongjia Zhang

**Affiliations:** ^1^Department of Cardiology, Beijing Anzhen Hospital, Capital Medical University, 100029 Beijing, China; ^2^Department of Nuclear Medicine, Beijing Anzhen Hospital, Capital Medical University, 100029 Beijing, China; ^3^Department of Cardiac Surgery, Beijing Anzhen Hospital, Capital Medical University, 100029 Beijing, China

**Keywords:** chronic coronary syndromes, magnetocardiography, myocardial perfusion imaging, machine learning algorithm

## Abstract

**Background:**

Magnetocardiography (MCG) is a novel non-invasive technique that detects subtle magnetic fields generated by cardiomyocyte electrical activity, offering sensitive detection of myocardial ischemia. This study aimed to assess the ability of MCG to predict impaired myocardial perfusion using single-photon emission computed tomography (SPECT).

**Methods:**

A total of 112 patients with chest pain underwent SPECT and MCG scans, from which 65 MCG output parameters were analyzed. Using least absolute shrinkage and selection operator (LASSO) regression to screen for significant MCG variables, three machine learning models were established to detect impaired myocardial perfusion: random forest (RF), decision tree (DT), and support vector machine (SVM). The diagnostic performance was evaluated based on the sensitivity, specificity, accuracy, positive predictive value (PPV), negative predictive value (NPV), and area under the receiver operating characteristic curve (AUC).

**Results:**

Five variables, the ratio of magnetic field amplitude at R-peak and positive T-peak (RoART+), R and T-peak magnetic field angle (RTA), maximum magnetic field angle (MAmax), maximum change in current angle (CCAmax), and change positive pole point area between the T-wave beginning and peak (CPPPATbp), were selected from 65 automatic output parameters. RTA emerged as the most critical variable in the RF, DT, and SVM models. All three models exhibited excellent diagnostic performance, with AUCs of 0.796, 0.780, and 0.804, respectively. While all models showed high sensitivity (RF = 0.870, DT = 0.826, SVM = 0.913), their specificity was comparatively lower (RF = 0.500, DT = 0.300, SVM = 0.100).

**Conclusions:**

Machine learning models utilizing five key MCG variables successfully predicted impaired myocardial perfusion, as confirmed by SPECT. These findings underscore the potential of MCG as a promising future screening tool for detecting impaired myocardial perfusion.

**Clinical Trial Registration:**

ChiCTR2200066942, https://www.chictr.org.cn/showproj.html?proj=187904.

## 1. Introduction

Coronary artery disease (CAD), characterized by atherosclerotic plaque deposition and luminal narrowing of the coronary arteries, is a leading cause of acute coronary syndromes (ACS) and chronic coronary syndromes (CCS), contributing significantly to global morbidity and mortality. The European Society of Cardiology (ESC) guidelines define CCS as a distinct phase of CAD, marked by stable plaques, vascular spasms, or microvascular diseases, and stable periods after ACS—excluding acute thrombotic ruptures [1]. Although coronary angiography (CAG) is recognized as the gold standard for diagnosing CAD, it is unsuitable for patients with CCS who lack obstructive CAD due to its invasiveness and the patient’s relative stability. Consequently, the ESC guidelines recommend a pre-test probability (PTP) of obstructive CAD based on age, sex, and symptoms [1]. Patients with PTP >15% are recommended to undergo non-invasive functional imaging to avoid excessive procedures and costs of these examinations [1]. Since anatomical stenosis of less than 90% of the luminal diameter is not necessarily associated with myocardial ischemia [2, 3], functional tests such as stress cardiac magnetic resonance (CMR), stress echocardiography, single-photon emission computed tomography (SPECT), and positron emission tomography (PET), rather than anatomical imaging such as coronary computed tomography angiography (CCTA), are required to determine the need for revascularization [1, 2, 3].

Myocardial perfusion imaging (MPI), including SPECT and PET, is regarded as the gold standard for non-invasive detection of abnormal perfusion and helps decision-making for follow-up treatment [4, 5]. These techniques can identify the region and extent of myocardial ischemia and differentiate ischemia from infarction by observing radiotracers extracted by cardiomyocytes at rest and during stress [4, 5]. Compared to initial CAG, MPI-guided therapeutic strategies have been associated with lower rates of revascularization, myocardial infarction (MI), and death in stable CAD patients [6]. However, MPI requires significant time investment, typically necessitating at least 4 h for complete inspection and analysis of results.

Magnetocardiography (MCG) is a novel noninvasive technique for detecting weak magnetic fields generated by the electrical activity of cardiomyocytes [7]. This technique is particularly useful when the heart experiences ischemia or infarction, leading to a reduction in blood flow and insufficient oxygen supply to cardiac cells [8, 9]. This shortage impacts the electrical activity of cardiomyocytes, causing abnormal cardiac depolarization or repolarization and altering the normal magnetic field map [10].

Myocardial ischemia or infarction can induce significant changes in magnetic fields, which are detectable and analyzable through MCG instruments. (1) A potential alteration is the multipolarization or unipolarization of the magnetic field: ischemic conditions may cause uneven electrical activity among cardiomyocytes, leading to a varied distribution of magnetic field intensity across different cardiac regions, typically exhibiting multipolarization patterns [11]. In cases of severe ischemia, unipolarization may occur when the magnetic field is significantly enhanced, primarily in one direction [12]. (2) Another change involves the deflection of the magnetic field angle, as myocardial ischemia can shift the orientation of cardiac electrical activity, causing variability in the direction of the magnetic field on the MCG [13]. (3) Additionally, an evident pole shift is observed due to the non-uniform electrical activity of cardiomyocytes, possibly relocating the heart’s magnetic field [14]. (4) Furthermore, myocardial ischemia may result in an abnormal or uneven distribution of the cardiac magnetic field, manifesting as an anomalous intensity distribution on MCG, which is indicative of injury and necrosis [15]. These alterations in the magnetic field can be directly detected and analyzed using MCG instruments.

Compared to an electrocardiogram (ECG), an MCG detects magnetic rather than electrical signals generated by the heart’s currents [8, 16]. This method exhibits superior stability as magnetic fields are not attenuated by the body and have minimal effect on contact resistance and muscle motion artifacts [8, 16]. Previous studies have shown that MCG is highly sensitive in diagnosing myocardial ischemia [12, 17, 18], and its superior ability to detect CAD has been validated in several clinical studies [19, 20]. Unlike SPECT, MCG does not require patients to be injected with tracers, and the entire scan can be completed within 90 seconds [7]. Therefore, MCG is emerging as a safe, feasible, convenient, and effective diagnostic tool for identifying impaired myocardial perfusion and can be particularly useful in diagnosing patients with CCS.

Testing with MCG currently involves two main approaches: image analysis and parameter determination. The images analyzed include magnetic field maps, pseudo-current density maps, and MCG waveforms [21, 22, 23]. Normally, in healthy individuals, the synchronization of cardiomyocyte electrical activity results in an organized magnetic dipole orientation without dispersion or splitting during the repolarization phase, as seen in magnetic field maps [7]. In contrast, myocardial ischemia results in biological injury currents and repolarization abnormalities, manifesting as dipole angulation or disorganization in magnetic field maps [7]. However, interpreting these images is time-consuming and subjective, requiring professional analysis. To address these challenges, there is a critical need to develop a predictive model that provides automatic output parameters, enhancing both diagnostic accuracy and efficiency.

Machine learning algorithms, including random forest (RF), decision tree (DT), and support vector machine (SVM), have become essential tools in medical data processing, especially for binary categorical variables [15, 16, 17, 18]. These algorithms have demonstrated exceptional capabilities and diagnostic accuracy in recent studies [24, 25, 26, 27]. In this study, we leveraged RF, DT, and SVM to establish three machine learning classifiers. The aim is to utilize selected MCG variables to predict abnormal myocardial perfusion as recognized by SPECT.

## 2. Methods

### 2.1 Participants

**Fig. 1.**
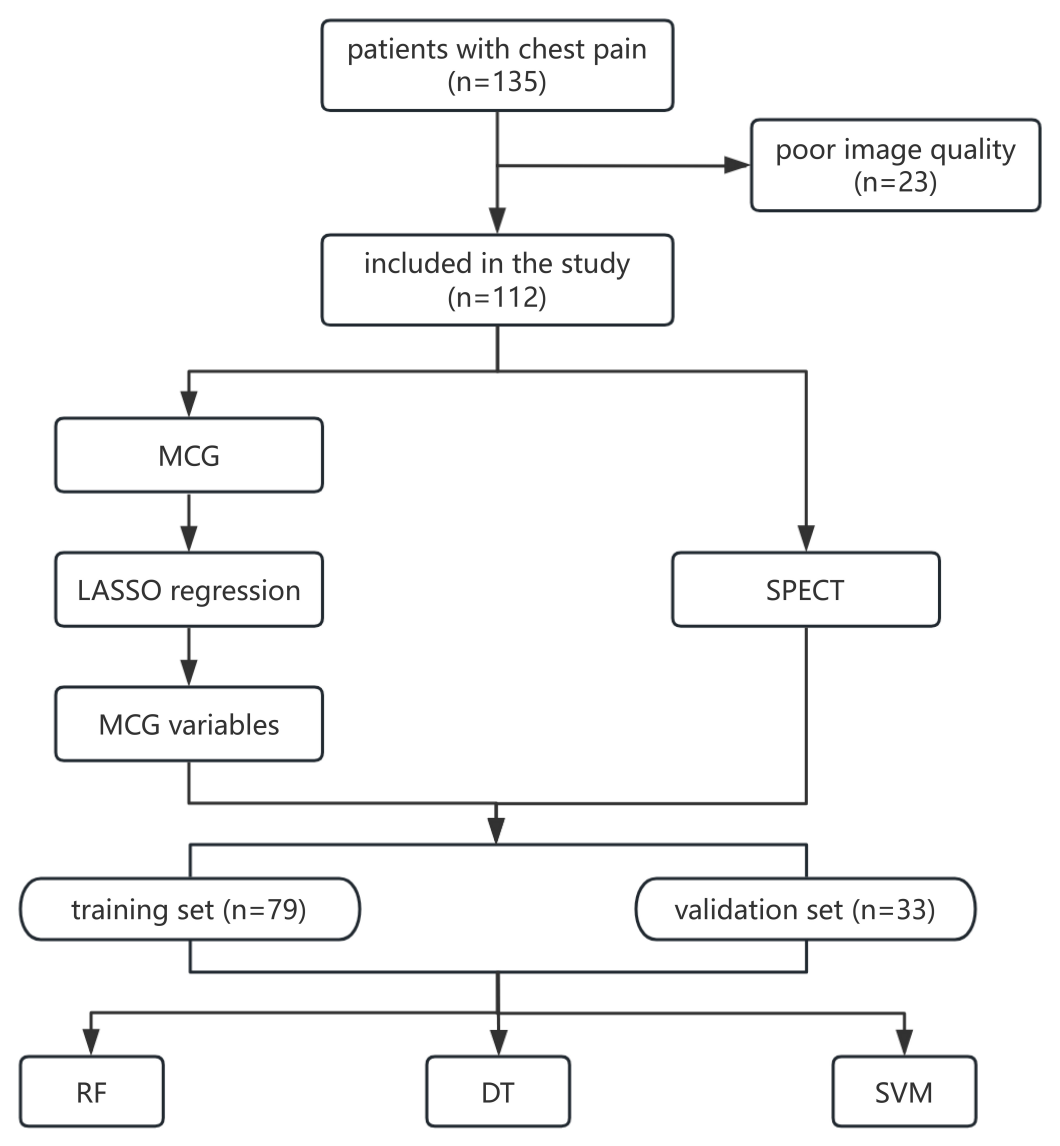
**Patient enrollment and analysis flowchart for evaluating myocardial perfusion using MCG and SPECT**. Initially, 135 outpatients with chest pain were enrolled in this study and 112 patients were included in the data analysis finally. These patients underwent SPECT testing and MCG recordings within a 3-month period before any revascularization procedures. Post-screening, significant MCG variables were identified, and three machine learning models (RF, DT, and SVM) were developed to detect reduced myocardial perfusion. Abbreviations: MCG, magnetocardiography; SPECT, single-photon emission computed tomography; RF, random forest; DT, decision tree; SVM, support vector machine; LASSO, least absolute shrinkage and selection operator.

This study was conducted at the Beijing Anzhen Hospital, Capital Medical University, and involved patients from the prospective cohort study ‘Application values of MCG in the CAD diagnosis’, which was approved by the Medical Ethics Committee of Beijing Anzhen Hospital affiliated to Capital Medical University (number: KS2022054). Informed consent was obtained from all participants. Initially, 135 patients with chest pain in an outpatient setting were enrolled. The exclusion criteria included the presence of magnetic implants (e.g., pacemakers, implantable cardioverter defibrillators), claustrophobia, New York Heart Association (NYHA) Class III/IV heart failure, severe chest deformities, or any other condition making MCG scans inappropriate. Due to size limitations, patient movement, and metal interference, 23 patients were excluded after inadequate MCG scans and sensor captures, leaving 112 patients (91 males and 21 females) in the data analysis. All patients underwent SPECT testing at rest and/or during stress as well as MCG recordings within 3 months before any revascularization. In this cohort, 70 patients were diagnosed with reduced myocardial perfusion by SPECT. A flowchart of the study is presented in Fig. 1. The study protocol conformed to the ethical guidelines of the 1975 Declaration of Helsinki as reflected in a priori approval by the institution’s human research committee.

### 2.2 Myocardial Perfusion Imaging Acquisition and Data Analysis

A conventional rest and exercise/drug stress two-day MPI imaging method was followed. Patients were instructed to discontinue theophyllines, nitrates, and β-receptor antagonists 1 to 2 days before the examination. During the stress test, adenosine (90 mg/30 mL) was injected intravenously at a rate of 0.14 mg⋅kg^-1^⋅min^-1^, Technetium 99m sestamibi (^99m^Tc-MIBI) was injected at the peak of adenosine administration, three minutes into the infusion, and MPI imaging was performed 1.5 h after injecting the imaging agent. The following day, resting MPI imaging was similarly performed 1.5 h after the intravenous injection of ^99m^Tc-MIBI in the resting state. Imaging was performed using a Siemens SYMBIA INTEVO 16 SPECT/CT machine, equipped with a SMARTZOOM collimator and IQ-SPECT technology for SPECT data acquisition and a computerized image reconstruction system. The images were analyzed according to the American Heart Association (AHA) guidelines, and the entire myocardium of the left ventricle was divided into 17 segments. A visual semi-quantitative 5-point scale was utilized for evaluation as follows: 0, normal radioactivity uptake; 1, mildly reduced radioactivity distribution; 2, moderately reduced radioactivity uptake; 3, severely reduced radioactivity uptake; and 4, radioactivity defects. Myocardial ischemia was defined as a segmental ‘reversible’ change in radioactivity distribution, whereas myocardial infarction was defined as a segmental ‘irreversible’ defect in radioactivity distribution in the myocardium.

### 2.3 MCG System and Recording

A 36-channel OPM-MCG system (Miracle MCG, Beijing X-MAGTECH Technologies Ltd., Beijing, China) was used for all MCG measurements. This system features a measurement sensitivity of <30 fT/Hz1/2 through the 36-channel atomic magnetometer. The direct-current residual magnetic field in the MCG measurement area was maintained below 5 nT. Notably, the system was operated in an examination room without the need for additional magnetic shielding. Prior to each measurement, all potential sources of magnetic field interference, including magnetic metal objects (e.g., metal dentures and glasses), electronic devices (e.g., mobile phones, watches, and electronic locators), and magnetic materials (e.g., magnets, bank cards, banknotes, and undergarments with magnetic components), were removed from the vicinity. Patients were placed supine on the examination bed, with the sensor array positioned approximately 2 cm above the chest. Controlled by the MCG system software, both the patient and the sensor array automatically entered the magnetic shielding space, initiating the automatic collection of cardiac magnetic field data. Each patient’s cardiac activity was continuously recorded across 36 points using an arrayed sensor in a 6 × 6 grid positioned above the chest, with a total recording time of 90 seconds.

### 2.4 MCG Data Analysis

After the MCG data collection was completed, the patient was automatically moved out of the magnetic shielding space, and the software automatically reconstructed high-precision magnetic field maps and pseudo-current density maps. These maps visually represented the spatiotemporal distribution of magnetic fields and provided outputs of the corresponding parameters. An independent investigator then conducted a quality evaluation and image analysis to exclude unqualified images. Sixty-five parameters associated with magnetic field, current angle, magnetic field amplitude, and magnetic pole change were collected from each patient.

### 2.5 Statistical Analysis

Data analyses were performed using IBM SPSS Statistics (version 26.0; IBM Corp., Armonk, NY, USA) and R 4.3.0 software (R Core Team, 2023, Vienna, Austria). The baseline data were expressed as mean value ± standard deviation (SD), with comparisons between mean values performed with an independent sample Student’s *t*-test to assess statistical significance between the ischemic and non-ischemic groups, assuming data normality. For non-normally distributed variables, the chi-square test was used. Variable selection was performed using least absolute shrinkage and selection operator (LASSO) regression to identify key MCG parameters. The study population was divided into a training set and a validation set in a 7:3 ratio, and machine learning models—RF, DT, and SVM—were developed using MCG variables [27, 28, 29, 30]. The RF model uses the random forest function of the random forest package (version 4.7-1.1), the DT model used the rpart function in the rpart package (version 4.1.23), and the SVM function of the e1071 package (version 1.7-14) was used for the SVM model. Diagnostic measures including sensitivity, specificity, positive predictive value (PPV), negative predictive value (NPV), and accuracy were calculated. Receiver operating characteristic (ROC) curves were plotted to calculate the areas under the ROC curves (AUC). Statistical significance was set at *p* < 0.05.

## 3. Results

### 3.1 Patient Demographics

Of the initial 135 patients, 112 met the inclusion criteria for the study. These patients had an average age of 58.93 ± 9.583 years (range: 35–77), with 91 (81.25%) being male. The median time between SPECT and MCG was 5.00 (2.00, 25.25) days. Impaired myocardial perfusion, indicative of myocardial ischemia or infarction, was observed in 70 patients. Notably, the prevalence of cardiovascular risk factors was significantly higher in this group, especially concerning sex, dyslipidemia, previous MI, and previous PCI. Specifically, in the group with impaired myocardial perfusion, 45 patients (64.3%) were diagnosed with hypertension, 24 (34.3%) with diabetes, and 62 (88.6%) with dyslipidemia. Comparatively, in the control group, these figures were 21 (50.0 %), 11 (26.2 %), and 30 (71.4 %), respectively. Among the 112 patients, 27 (38.6%) patients in the experimental group and 9 (21.4%) patients in the control group had a history of smoking. Twenty-four (34.3%) patients in the experimental group were diagnosed with MI, and 21 (30%) underwent PCI (Table 1).

**Table 1.** **Comprehensive demographics, cardiovascular risk factors, and therapeutic interventions for patients with and without impaired myocardial perfusion**.

	Impaired myocardial perfusion	Normal myocardial perfusion	*p* value
Men, n (%)	65 (92.9%)	26 (61.9%)	0.000
Age, years	58.90 ± 9.97	58.98 ± 9.01	0.968
Hypertension, n (%)	45 (64.3%)	21 (50.0%)	0.137
Diabetes, n (%)	24 (34.3%)	11 (26.2%)	0.371
Dyslipidemia, n (%)	62 (88.6%)	30 (71.4%)	0.022
Smoking, n (%)	27 (38.6%)	9 (21.4%)	0.060
Previous MI, n (%)	24 (34.3%)	6 (14.3%)	0.021
Previous PCI, n (%)	21 (30%)	4 (9.5%)	0.012

Values are n (%) or mean ± SD. Abbreviations: MI, myocardial infarction; PCI, percutaneous coronary intervention; SD, standard deviation.

### 3.2 LASSO Regression for MCG Variable Selection

**Fig. 2.**
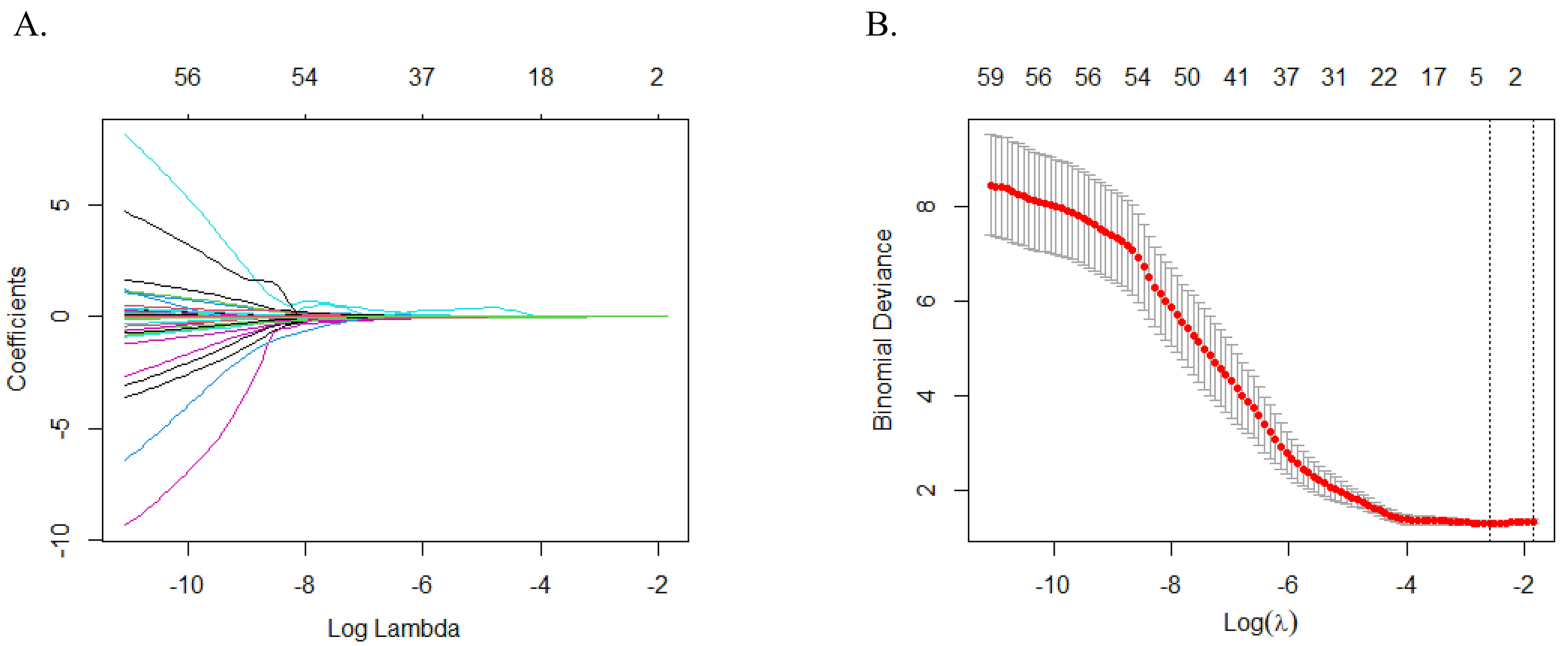
**LASSO regression analysis for MCG variable selection**. (A) The variation characteristics of the coefficient of variables. (B) The selection process of the optimum value of the parameter λ in the LASSO regression model by cross-validation method. Abbreviations: LASSO, least absolute shrinkage and selection operator; MCG, magnetocardiography.

To optimize the selection of meaningful MCG variables, we utilized the LASSO selection method with 10-fold cross-validation. The variation in characteristics for these variables is depicted in Fig. 2A. We determined 0.061440 as the optimal regularization parameter, which was lambda.min (shown in Fig. 2B), chosen specifically to minimize prediction errors. This parameter was used to select five key variables—the ratio of magnetic field amplitude at R-peak and positive T-peak (RoART+), R and T-peak magnetic field angle (RTA), maximum magnetic field angle (MAmax), maximum change in current angle (CCAmax) and change positive pole point area between T-wave beginning and peak (CPPPATbp)—due to their strong association with myocardial perfusion (Table 2). These variables will serve as crucial inputs in the predictive model.

**Table 2.** **Key MCG parameters identified by LASSO regression for myocardial perfusion analysis**.

Parameter	Definition
RoART+	The ratio of magnetic field amplitude at R-peak and the positive amplitude at T-peak
RTA	The magnetic field angle between R-peak and T-peak
MAmax	The maximum magnetic field angle at intervals of a certain time τ within TT segment
CCAmax	The maximum value of changes in current angle at intervals of a certain time τ within TT segment
CPPPATbp	The change in positive pole point area between T-begin and T-peak

Abbreviations: RoART+, the ratio of magnetic field amplitude at R-peak and positive T-peak; RTA, R and T-peak magnetic field angle; MAmax, maximum magnetic field angle; CCAmax, maximum change in current angle; CPPPATbp, change positive pole point area between T-wave beginning and peak; LASSO, least absolute shrinkage and selection operator; MCG, magnetocardiography.

### 3.3 RF, DT and SVM Models for Prediction of Abnormal Myocardial Perfusion

**Fig. 3.**
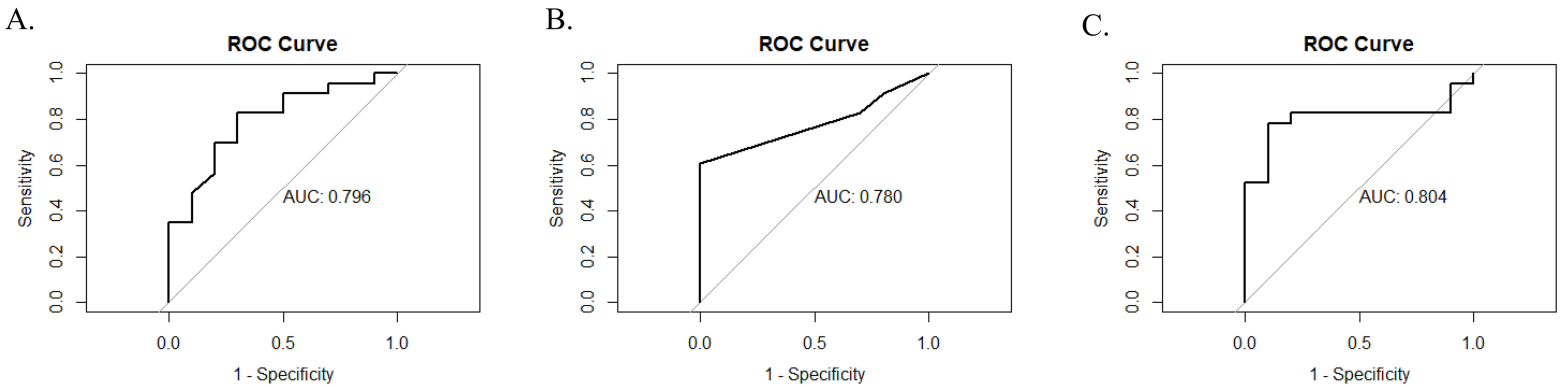
**ROC curves and AUC values for RF, DT and SVM models**. (A) RF. (B) DT. (C) SVM. These graphs highlight the diagnostic performances of the models with their respective AUC values of 0.796, 0.780, and 0.804. The models demonstrated high sensitivity (RF = 0.870, DT = 0.826, and SVM = 0.913) but varied in specificity (RF = 0.500, DT = 0.300, and SVM = 0.100). Abbreviations: ROC, receiver operating characteristic; AUC, area under the receiver operating characteristic curve; RF, random forest; DT, decision tree; SVM, support vector machine.

After confirming the five variables through LASSO regression, we developed three predictive models, RF, DT, and SVM, to assess if the MCG variables predict impaired myocardial perfusion. All included participants were randomly assigned to a training set (n = 79) or a validation set (n = 33) in a ratio of 7:3. Each model utilized the five screened MCG variables: RoART+, RTA, MAmax, CCAmax, and CPPPATbp. According to the ESC Guidelines, impaired myocardial perfusion was defined as exhibiting either a reversible or fixed deficit on SPECT [1, 31]. The diagnostic performances of the three models in the validation set are presented in Table 3 and Fig. 3. All three models demonstrated excellent diagnostic accuracy, with AUC values of 0.796, 0.780, and 0.804, respectively. Additionally, the models showed high sensitivity (RF = 0.870, DT = 0.826, and SVM = 0.913), whereas their specificity was comparatively lower (RF = 0.500, DT = 0.300, and SVM = 0.100). In terms of predictive performance, the RF model had a positive predictive value (PPV) of 0.800, a negative predictive value (NPV) of 0.625, and an overall accuracy of 0.758. The accuracies of the DT and SVM models were 0.667, PPVs of 0.730 and 0.700, and NPVs of 0.428 and 0.333, respectively.

**Table 3.** **Comparative diagnostic performance of RF, DT, and SVM models in the validation set**.

Models	Performance					
	Sensitivity	Specificity	PPV	NPV	Accuracy	AUC
RF	0.870	0.500	0.800	0.625	0.758	0.796
DT	0.826	0.300	0.730	0.428	0.667	0.780
SVM	0.913	0.100	0.700	0.333	0.667	0.804

This table compares the diagnostic performance of three machine learning models—RF, DT, and SVM—in the validation set. Performance metrics include Sensitivity, Specificity, PPV, NPV, Accuracy, and AUC. Abbreviations: RF, random forest; DT, decision tree; SVM, support vector machine; PPV, positive predictive value; NPV, negative predictive value; AUC, area under the receiver operating characteristic curve.

**Fig. 4.**
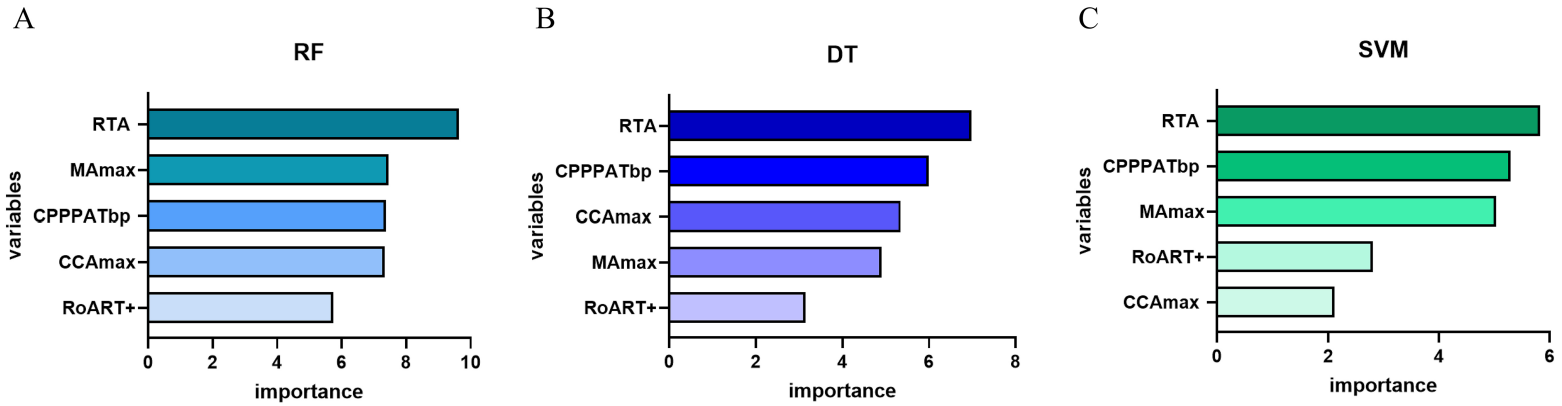
**The ranking of variables in all three models**. (A) RF. (B) DT. (C) SVM. This figure illustrates the importance of five variables within three predictive models. RTA emerged as the most crucial variable across all models, significantly influencing the model outcomes. Abbreviations: RF, random forest; DT, decision tree; SVM, support vector machine; RTA, R and T-peak magnetic field angle; MAmax, maximum magnetic field angle; CPPPATbp, change positive pole point area between T-wave beginning and peak; CCAmax, maximum change in current angle; RoART+, the ratio of magnetic field amplitude at R-peak and positive T-peak.

As shown in Fig. 4, the importance of each variable was determined using the calculated coefficients in each model. Among the five parameters evaluated, RTA emerged as the most critical variable in the RF, DT, and SVM models. In contrast, the importance of the remaining parameters varied between each model. Specifically, CPPPATbp was identified as the second most significant variable in the DT and SVM models, while MAmax was ranked second in the RF model.

Additionally, 41 patients with impaired SPECT myocardial perfusion underwent CAG within 3 months of SPECT and MCG testing. Of these, 28 patients underwent revascularization during the CAG procedure. Using a criterion of stenosis ≥50% for defining significant CAD, 35 patients were identified with positive CAG results, and 6 with negative results. Both the SPECT and MCG tests showed positive outcomes in these patients. Therefore, with CAG as the gold standard, the sensitivities of both SPECT and MCG were determined to be 1.00, indicating perfect agreement in detecting clinically relevant coronary obstructions.

## 4. Discussion

To the best of our knowledge, this is the first study to explore the correlation between OPM-MCG and SPECT in an unshielded clinical environment. We analyzed 65 MCG parameters and selected five critical variables using LASSO regression. Subsequently, we employed three machine learning algorithms—RF, DT, and SVM—to develop predictive models. These models demonstrated exceptional diagnostic performance in detecting impaired myocardial perfusion, as identified by SPECT. We further evaluated the importance of each variable across all three models and found that RTA, which characterizes the magnetic field’s properties, was the most influential variable.

Unlike prior MCG research, this study was conducted in an unshielded clinical setting. The magnetic fields generated by myocardial cell activity are exceedingly weak, approximately 10^-11^ to 10^-14^ T, compared with Earth’s magnetic field of about 10^-4^ T [32]. Traditionally, MCG assessments required magnetically shielded rooms to mitigate environmental magnetic noise [33, 34, 35]. However, the OPM-MCG used in our study integrates built-in shielding within the device itself, providing robust anti-interference capabilities against environmental magnetic fields. This study successfully demonstrates that high-quality MCGs scans, including magnetic field maps and pseudo-current density maps, can be performed in unshielded environments using the advanced OPM-MCG, thus supporting the feasibility of broader clinical application without the need for specialized shielding infrastructure.

According to the 2019 ESC of Cardiology guidelines, functional stress imaging is highly recommended for patients with CCS who exhibit new symptoms after PTP evaluation to guide further treatment strategies [1]. However, the use of tracers and the associated waiting time for tracer distribution are both time-consuming and inconvenient. In contrast, MCG, which has been proven to be highly sensitive to ischemia, overcomes these drawbacks. It is likely to effectively identify impaired myocardial perfusion and can thus be applied in diagnosing patients with CCS. A previous study showed that MCG’s performance in detecting significant CAD, as diagnosed by CAG, was not inferior to that of stress SPECT [36]. In this study, we further explored the capacity of MCG to identify impaired myocardial perfusion, as confirmed using SPECT, highlighting its potential as an efficient and non-invasive diagnostic tool.

By analyzing the diagnostic indicators, we found that all three models exhibited high sensitivity but had relatively lower specificity. Several factors contribute to this low specificity in MCG. Primarily, patients with a history of diabetes who present with positive MCG results but negative SPECT results may exhibit coronary microvascular dysfunction (CMD), which is prevalent in this demographic [37, 38, 39]. Although PET scans, which measure absolute quantitative myocardial blood flow (MBF) and coronary flow reserve (CFR), remain the gold standard for noninvasively assessing CMD, quantifying CFR in SPECT is challenging due to its lower sensitivity [40, 41, 42]. In our study, myocardial perfusion was evaluated using a visual semi-quantitative 5-point scale, which may not effectively recognize coronary microvascular lesions. However, MCG has the capability to detect subtle magnetic field changes caused by abnormal electrical activity of cardiomyocytes, potentially identifying coronary microvascular ischemia that is not detected by SPECT.

In our study, 26.2% of patients who exhibited normal myocardial perfusion on SPECT (11/42) had diabetes, suggesting that CMD should explain some of the low specificity observed with MCG. However, the diagnosis of CMD mainly relies on the measurement of the index of microvascular resistance (IMR) during CAG or CFR using PET—procedures that were not performed on our study participants. Consequently, further research is necessary to validate our hypothesis that the diagnostic capabilities of MCG for CMD can effectively assess microvascular dysfunction.

Additionally, SPECT reflects the relative blood flow in the myocardium. When lesions are uniformly present across all three coronary artery branches, balanced ischemia may occur, potentially leading to false-negative results in SPECT imaging. In situations where patients exhibit severe symptoms yet present with normal SPECT results, MCG can be an invaluable supplementary diagnostic tool to assess the need for further CAG. Because of these dynamics, MCG often yields a higher number of false positives and fewer true negatives, reflecting its lower specificity and enhanced sensitivity. Therefore, MCG has proven to be an effective screening method for myocardial ischemia, adept at identifying patients at high risk for impaired myocardial perfusion. For patients with positive MCG results, additional diagnostic evaluations are recommended to mitigate the risk of overlooked ischemia.

In our study, the RTA, which measures the magnetic field angle between the R-peak and T-peak, emerged as the most critical parameter across all three models. This finding aligns with several studies that have identified the magnetic field angle as a fundamental parameter for the diagnosis of myocardial ischemia. For instance, Ramesh *et al*. [21] investigated 29 patients with chest pain and normal resting ECG s, reporting that an abnormal magnetic field angle was significantly more prevalent among patients with positive treadmill test results (72% vs. 6%). Similarly, Lim *et al*. [43] evaluated 24 MCG parameters and identified the field-map angle as one of the most sensitive for detecting patients with non-ST-elevation myocardial infarction (NSTEMI). Our results corroborate these earlier findings, underscoring the importance of changes in the magnetic field angle—caused by cardiomyocyte injury—in the detection of myocardial ischemia.

This study had several limitations. First, as a single-center study, the robustness and generalizability of our findings, including the MCG parameters and machine learning models, cannot be conclusively determined. Subsequent multicenter studies are required to apply these parameters and models to validate their performance across broader populations. Second, the sample size was relatively small, reflecting the limited routine use of SPECT in clinical practice. Again, larger studies are needed to construct more stable and reliable models to aid in the diagnosis of myocardial ischemia. Finally, patients were categorized based solely on the presence or absence of reduced myocardial perfusion detected by SPECT, without considering the specific degrees and locations of decreased perfusion. In future studies, we intend to increase the number of enrolled patients and construct models to predict the degree and location of impaired myocardial perfusion.

## 5. Conclusions

This study demonstrates that machine learning models—specifically RF, DT, and SVM—possess excellent data processing capabilities, making them highly effective for detecting impaired myocardial perfusion. Notably, the R and T-peak magnetic field angle (RTA) emerged as the most critical variable in all the three models. Consequently, MCG shows great potential as an effective screening tool for identifying patients at high risk of impaired myocardial perfusion. Looking forward, MCG could significantly enhance diagnostic strategies for myocardial ischemia.

## Availability of Data and Materials

The datasets analyzed in this study are available from the corresponding author upon reasonable request.
